# Behavioral Changes Associated With COVID-19 Vaccination: Cross-National Online Survey

**DOI:** 10.2196/47563

**Published:** 2023-10-31

**Authors:** Alessandro De Gaetano, Paolo Bajardi, Nicolò Gozzi, Nicola Perra, Daniela Perrotta, Daniela Paolotti

**Affiliations:** 1 ISI Foundation Turin Italy; 2 Aix Marseille Univ, Université de Toulon, CNRS, CPT Marseille France; 3 CENTAI Institute Turin Italy; 4 Networks and Urban Systems Centre University of Greenwich London United Kingdom; 5 School of Mathematical Sciences Queen Mary University of London London United Kingdom; 6 Laboratory of Digital and Computational Demography Max Planck Institute for Demographic Research Rostock Germany

**Keywords:** COVID-19, vaccines, social behaviors, online surveys, nonpharmaceutical interventions, survey, vaccination, behavior, NPIs, prevention

## Abstract

**Background:**

During the initial phases of the vaccination campaign worldwide, nonpharmaceutical interventions (NPIs) remained pivotal in the fight against the COVID-19 pandemic. In this context, it is important to understand how the arrival of vaccines affected the adoption of NPIs. Indeed, some individuals might have seen the start of mass vaccination campaigns as the end of the emergency and, as a result, relaxed their COVID-safe behaviors, facilitating the spread of the virus in a delicate epidemic phase such as the initial rollout.

**Objective:**

The aim of this study was to collect information about the possible relaxation of protective behaviors following key events of the vaccination campaign in four countries and to analyze possible associations of these behavioral tendencies with the sociodemographic characteristics of participants.

**Methods:**

We developed an online survey named “COVID-19 Prevention and Behavior Survey” that was conducted between November 26 and December 22, 2021. Participants were recruited using targeted ads on Facebook in four different countries: Brazil, Italy, South Africa, and the United Kingdom. We measured the onset of relaxation of protective measures in response to key events of the vaccination campaign, namely personal vaccination and vaccination of the most vulnerable population. Through calculation of odds ratios (ORs) and regression analysis, we assessed the strength of association between compliance with NPIs and sociodemographic characteristics of participants.

**Results:**

We received 2263 questionnaires from the four countries. Participants reported the most significant changes in social activities such as going to a restaurant or the cinema and visiting relatives and friends. This is in good agreement with validated psychological models of health-related behavioral change such as the Health Belief Model, according to which activities with higher costs and perceived barriers (eg, social activities) are more prone to early relaxation. Multivariate analysis using a generalized linear model showed that the two main determinants of the drop of social NPIs were (1) having previously tested positive for COVID-19 (after the second vaccine dose: OR 2.46, 95% CI 1.73-3.49) and (2) living with people at risk (after the second vaccine dose: OR 1.57, 95% CI 1.22-2.03).

**Conclusions:**

This work shows that particular caution has to be taken during vaccination campaigns. Indeed, people might relax their safe behaviors regardless of the dynamics of the epidemic. For this reason, it is crucial to maintain high compliance with NPIs to avoid hindering the beneficial effects of the vaccine.

## Introduction

The COVID-19 pandemic impacted humanity on an unprecedented scale with, as of February 2023, over 757 million confirmed cases causing over 6.8 million deaths [1]. At the beginning of 2021, vaccination campaigns were rolled out in many countries, providing a pharmaceutical measure to protect against the most severe manifestations of the disease and to contrast the spreading of the virus. Before vaccines were made available, the mitigation of infections and deaths was largely achieved through nonpharmaceutical interventions (NPIs) such as lockdowns, social distancing, curfews, and use of protective face masks [2]. These measures aimed at controlling the epidemic diffusion by reducing overall social contacts as well as by limiting the spreading potential of unavoidable social interactions [3-5]. A significant body of literature focused on the efficacy of these measures in reducing disease transmission across different contexts and geographies [6-9] and the socioeconomic disruption to everyday life brought by stringent NPIs and their unequal impact on the population [10-13]. Despite the incredible milestone in the fight against SARS-CoV-2 represented by the start of vaccination campaigns worldwide, due to the initial limited supply and unprecedented logistic challenges, NPIs remained essential, at least in the first phases, to sustain the efforts of mass immunization campaigns and to reach adequate vaccination coverage [14,15].

In this complex context, the interplay between population-level mitigation measures, individual decisions related to adoption of these measures, and the vaccination remains relatively less explored. Previous studies have focused, primarily from a mathematical modeling standpoint, on the interplay between NPIs adoption, COVID-19 spread, and vaccination campaigns [15-18]. They have shown that early relaxation of COVID-safe behaviors may contribute to further avoidable infections and threaten the success of vaccination efforts. Nonetheless, empirical evidence to support and quantify if and at which rate individuals relax their behavior in response to the COVID-19 vaccination is still very limited [19].

In this study, we tackled this limitation by studying, from a data-driven standpoint, how individual vaccination status and national rollout advancement impacted the adoption of protective behaviors such as hand washing, mask wearing, and social distancing. To account for different national contexts, especially related to the heterogeneity of vaccination campaigns’ progress and to the COVID-19 epidemiological situation worldwide, we developed a cross-country survey that we administered to a random sample of anonymous individuals targeted through the advertisement platform of Facebook in Brazil, Italy, South Africa, and the United Kingdom. These four countries were selected based on the diversity of the epidemiological situation, vaccination coverage, and NPIs implementation at the time when the survey was conducted to obtain more general and solid results. More details in this regard are provided in the Methods section.

The use of targeted Facebook ads to collect relevant social data has become a frequent practice in the field of computational social science [20,21]. This was particularly true during the COVID-19 pandemic, when the dissemination of epidemiological and behavioral surveys through the Facebook advertisement platform gained significant traction. One of the earliest and most successful examples is the COVID-19 World Symptoms Survey [22] that was deployed from March 2020 to June 2022, in partnership with University of Maryland and Carnegie Mellon University, to collect data about COVID-19 vaccine acceptance, preventive behaviors, and symptoms. The many insights provided by these studies were crucial to demonstrate the value of online surveys for tracking patterns and trends in COVID-19 outcomes in a complementary fashion with respect to official reporting [23,24].

Our work falls within this line of research and is aimed at measuring individual behavioral changes (eg, adoption or relaxation of protective measures) in association with different stages of the vaccination campaign. To account for nonrepresentative sampling, we utilized a poststratification weighting method that is commonly used in survey research. This approach helps to approximate a representative sample of the population in each country. Specifically, in our survey, we asked about the compliance with NPIs related to six different activities after key events of the vaccination campaign, namely the vaccination of older adults and people at risk and the personal inoculation of the first and second doses. Although our study did not include a comparison of behaviors before and after vaccination, our questions were crafted to evaluate self-reported changes in behaviors during these particular stages of the vaccination campaign. Rather than inquiring about specific protective measures in separate data collection periods before and after vaccination, we asked participants if they had relaxed the protective measures they had been taking following critical moments in the vaccination campaign. This approach enabled us to determine how the adoption of NPIs changed in relation to specific vaccination events. By studying the over 2000 responses received, we found that NPIs related to social behaviors were those that were relaxed the most after key stages of the vaccination campaign. This is in good agreement with the constructs of psychological theoretical frameworks such as the Health Belief Model (HBM), which suggests that NPIs with higher associated costs are more difficult to adopt and thus are generally the first to be relaxed. From this standpoint, we performed a multivariate analysis using a generalized linear model (GLM) to quantify the association between the relaxation of social behaviors and sociodemographic characteristics of respondents, such as country of residence, age group, and sex. Our results show that the two most important determinants associated with the relaxation of social behaviors are having tested positive for COVID-19 and living with people at risk.

## Methods

### Study Context

**Figure 1:**
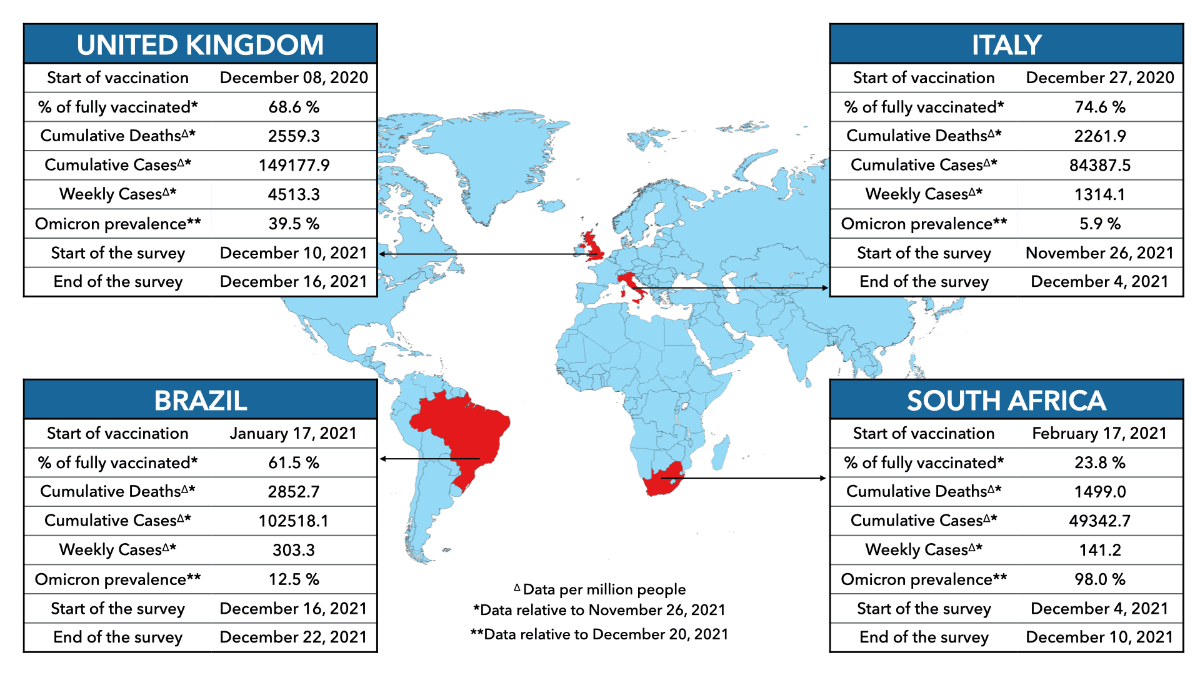
Informative data about each country included in the study [25] with the dates of the beginning of the advertising campaigns.

Our survey study was conducted during late November/December 2021. As of December 1, 2021, Italy and the United Kingdom had respectively 75% and 69% of individuals that completed the initial COVID-19 vaccination protocol. Vaccination uptake was slightly lower in Brazil (62%), while only 24% of people were fully vaccinated in South Africa [25]. In December 2021, a new and more transmissible SARS-CoV-2 variant of concern (VOC) emerged: B.1.1.529 (Omicron). In mid-December 2021, Omicron was mostly dominant in South Africa (95% of sequenced genomes) and a steep increase in the number of cases was observed in the United Kingdom (39%) due to this VOC. However, in Brazil and Italy, Omicron prevalence was still much lower (12% and 5%, respectively), but was quickly increasing. These details are illustrated in Figure 1 together with additional epidemiological indicators (eg, cumulative number of COVID-19 cases) in the surveyed countries.

### Study Design

The questionnaire “COVID-19 Vaccines and Behaviors Survey” that we developed consisted of two sections. The first aimed at collecting sociodemographic features such as sex and age. The second focused on understanding to what extent individuals changed behaviors in response to the COVID-19 vaccine rollout and which behaviors they relaxed, if any. We considered three different pivotal stages of the vaccination campaign that may have acted as a trigger for the behavior change of individuals: vaccination of those at higher risk of severe symptoms following COVID-19 infection (those aged 65+ years and people with comorbidities), the start of the individual vaccination cycle (ie, the first dose), and the end of the individual vaccination cycle (ie, the second dose). Indeed, since the outcome of COVID-19 infections strongly depends on age and on medical condition, people may have decided to drop COVID-safe behaviors once the population at higher risk had been immunized. Similarly, individuals may have felt reassured by personal (partial and full) immunization and adapted their behaviors accordingly. We conducted our survey study in 4 countries that we selected based on different characteristics in vaccination coverage, disease prevalence, and dominant SARS-CoV-2 variant: Brazil, Italy, South Africa, and the United Kingdom. The master version of the survey was created in English and then translated, with the help of native speakers, in Italian and Portuguese. The survey was implemented via Limesurvey [26], a web app that allows the easy deployment of surveys at scale. The English version of the questionnaire is provided in Multimedia Appendix 1.

To recruit participants, we distributed the survey via targeted Facebook advertisements created using Facebook Ads Manager [27]. We followed the methodology illustrated by Pötzschke and Braun [28], and more recently by Perrotta et al [29]. We created a separate advertising campaign for each country. Each campaign contained one ads set for each combination of the three targeting variables used to stratify the population: sex (male, female), age (18-24, 25-44, 45-64, 65+ years), and region of residence (based on statistical division of each country; a complete list of the macro regions used is provided in Table S1 of Multimedia Appendix 2). These are well-known confounding variables and, through the stratification of ads delivery, we were able to obtain sufficient responses in each stratum. Following this approach, we obtained 32 strata for South Africa and 40 each for Brazil, Italy, and the United Kingdom. Each of them contained 6 ads that differed only in the ad image used. In contrast to the description by Pötzschke and Braun [28], Facebook no longer allows more than 250 active ads at the same time. For this reason, we launched the advertising campaign for each country separately, starting with Italy on November 26, 2021, and ending with Brazil on December 22, 2021, as shown in Figure 1. More details on the survey methodology are provided in Section S1 of Multimedia Appendix 2.

### Inclusion Criteria

We collected a total of 2263 responses: 435 from Italy (19.2% of total), 386 from the United Kingdom (17.1%), 305 from Brazil (13.5%), 1014 from South Africa (44.8%), and 123 (5.4%) submitted from other countries or with no answers (that we discarded). We included in our analysis only questionnaires containing information about sex, age, and region of residence, as we needed these features to correct for nonrepresentativeness of the sample. This led to the exclusion of 196 questionnaires (8.66% of the total). In the multivariate analysis presented below, we used additional features, namely education attainment, household composition, vaccination status, previous positivity to SARS-CoV-2, and presence of risk factors for COVID-19. Therefore, for this analysis, we excluded questionnaires in which these features were missing (22% of the total). Additional information on respondent selection is reported in Section S2 of Multimedia Appendix 2.

After the exclusion of noneligible respondents, we applied poststratification weights to align our samples with the general population of the countries considered. We stratified our respondents by sex, age, and region, and we calculated the percentage of respondents (sp_k_) in each stratum k. We then computed the actual percentage of the population in each stratum (rp_k_). We used data from World Population Prospects [30], which provided estimates of the population in each country in 2020, divided by sex and 5-year age groups. This subdivision of age groups did not allow for direct comparison with survey counts for the 18-24 years age group. To address this problem, we summed the actual population in the 20-24 years age group with 2/5 of the population stated for the 15-19 years age group. Finally, we computed the poststratification weight for each stratum as the ratio between the two percentages: rp_k_/sp_k_.

### Ethical Approval

Ethical approval was obtained from the Bioethical Committee of the University of Turin (280342 del 8.5.2021).

### Measuring Behavioral Changes

As a proxy for reduced adoption of COVID-safe behaviors, we considered the following six possible changes in activities: (1) using public transport more frequently, (2) engaging in social activities more frequently (eg, going to restaurants), (3) visiting relatives and friends more frequently, (4) reducing hygiene measures (eg, wash hands less often, use disinfectant gel less often), (5) wearing a face mask less often (where not mandatory), and (6) reducing the recommended physical distance (1 or 2 meters) from other people. We chose these activities as representatives of the main NPIs that were widely implemented to contrast the spread of SARS-CoV-2 before the arrival of vaccines. Specifically, survey respondents were asked if they felt more comfortable doing these activities after each of the three trigger stages of the vaccination campaign mentioned above. Possible answers to these questions were organized using a 5-point Likert scale (1=definitely not, 2=no, 3=neither yes nor no, 4=yes, and 5=definitely yes). Participants were also given the opportunity to report “not applicable” as an answer. These cases were treated as missing values. See Table S2 of Multimedia Appendix 2 for more information.

For each activity, we transformed responses into a binary variable defining whether an individual engaged or not in behavior change related to that activity. In particular, options 4 (yes) and 5 (definitely yes) were associated with a change in behavior, whereas options 1 (definitely no), 2 (no), and 3 (neither yes nor no) were associated with no change.

We also investigated a hypothetical scenario in which we asked respondents about their potential behavioral reaction in case of a future worsening of epidemiological conditions. In this case, we considered four measures they could adopt: (1) wear a face mask more frequently (where not mandatory), (2) reduce social contacts, (3) keep a greater physical distance from other people, and (4) avoid crowded places. Responses were also given on a 5-point Likert scale (from 1=very unlikely to adopt to 5=very likely to adopt), and we considered that a single NPI would be adopted with response options 4 (likely) and 5 (very likely) and not adopted with response options 1 (very unlikely), 2 (unlikely), and 3 (neutral). Again, “not applicable” answers were treated as missing values.

### Multivariate Analysis

Additionally, we investigated how the relaxation of NPIs related to social behaviors was associated with respondents’ social and demographic characteristics. We focused on social behaviors since, according to the HBM framework, they are generally perceived as more costly to give up and thus relaxation of NPIs in these settings is more likely to occur. The HBM is a well-known social and psychological model regarding the adoption of health-related behaviors [31-33], which posits that the risk perception of an individual plays a pivotal role in the adoption of health behaviors. In detail, two constructs contribute to the individual perceived threat: individuals’ belief on how likely they are to contract the disease (perceived susceptibility) and personal evaluation of the severity of the consequences of the disease (perceived severity). The perceived threat can lead to the adoption (or the relaxation) of a health-related behavior. Additionally, individuals ground their choices on perceived benefits and perceived barriers. Perceived benefits associated with a health behavior are the personal opinions on the value or usefulness of that behavior in reducing the perceived threat, whereas perceived barriers are the individual’s perception of the obstacles of adopting the new behavior. This implies that, for example, health-related behaviors with high perceived benefits may not be adopted because of their high perceived barriers. As a result, health behaviors with high perceived barriers are usually the first to be relaxed. NPIs regarding social behaviors, despite having high impact on reducing the spread of the disease, are associated with a high perceived cost as they prevent individuals from participating in everyday activities and from interacting with their families or friends. For this reason, of the six activities included in our questionnaire, in this part of the analysis, we focused our attention only on “Engage in social activities more frequently (eg, going to restaurants)” and “Visit relatives and friends more frequently,” which will hereafter be referred to collectively as “social behaviors”.

For the multivariate analysis, we used a fixed-effects model with a logistic regression and a binary outcome of 1 if at least one social behavior was changed and 0 otherwise. To consider poststratification weights in the regression, we opted for the GLM available in the Python library Statsmodels [34]. We used a logit function and a binomial function for outcome. In this way, the GLM is effectively a logistic regression with weights. We chose logistic regression because of its explainability of the coefficients. Nonetheless, we tested other models for comparison (see Multimedia Appendix 2 for details). The features included in the model are provided in Table 1 with the corresponding reference values. Moreover, we transformed each categorical feature in a set of dummy variables, using the most frequent as a reference. For example, the feature age, which can take the values 18-24, 25-44, 45-64, and 65+, was encoded into 3 dummy variables (age 18-24, age 25-44, age 45-64), considering the age group 65+ as the reference. See Section S3 and Table S3 in Multimedia Appendix 2 for an analysis on the independence of the features and Table S4 of Multimedia Appendix 2 for the robustness analysis.

**Table 1:** Variables included in the multivariate analysis and their values.

Variable	Values
Gender	Female (reference), male
Age group (years)	18-24 (reference), 25-44, 45-64, 65+
Country	Italy, South Africa (reference), United Kingdom, Brazil
Risk	Yes (have a risk factor for COVID-19, such as respiratory chronic diseases or immunocompromised state), No (reference)
Risk in household	Yes (have a person in household with a risk factor for COVID-19, such as respiratory chronic diseases or immunocompromised state), No (reference)
<18 in household	Yes (have at least one household member who is under 18 years old), No (reference)
>65 in household	Yes (have at least one household member who is above 65 years old), No (reference)
Positive test	Yes (previously tested positive for COVID-19), No (reference)
Vaccine	Yes (received at least one dose of vaccine), No (reference)

The model is ruled by the following equation:



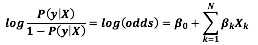



where *P(y|X)* is the conditional probability of the binary outcome (*y*) given the set of features (*X*), β_0_ is the intercept of the model, and β_k_ is the coefficient related to each feature X_k_. If we consider, for example, X_1_ to be the binary variable age 18-24, then the coefficient β_1_ is the logarithm of the odds ratio (OR) comparing age group 18-24 with the reference group 65+. The OR is often used in epidemiology to assess the strength of an association between an outcome and an exposure. In particular, it represents the ratio between the odds of the outcome in the presence of the exposure and the odds of the outcome in the absence of the exposure. Following the example above, the exponential of β_1_ is the ratio of the odds of changing social behaviors (ie, the outcome) if being part of the age group 18-24 (ie, the exposure) divided by the odds of changing social behaviors if being part of the age group 65+ (ie, the value taken as a reference). Therefore, by obtaining the exponential of the coefficients from the multivariate analysis, we immediately obtained a measure of the association between the variable we are considering and the change in social behavior.

The analysis was performed for all three stages of the vaccination campaign considered in this study: vaccination for those over 65 years old and people with comorbidities (ie, the groups at risk), personal first dose, and second dose. While the survey question related to the first event was accessible to all respondents, the questions related to the first and second doses were only available to vaccinated people. Nonetheless, these represented almost 80% of the respondents; therefore, the sample can be considered to be mostly the same.

For the scenario related to a worsening of the epidemiological conditions, we performed a similar analysis. We included the same features but, in this case, the binary outcome was 1 if all four NPIs would be readopted and 0 otherwise.

## Results

### Sample Composition

Table 2 shows the demographic characteristics of the sample by country, before applying poststratification weights. These numbers exclude the data of participants that did not report age, sex or country, but include the data of participants not reporting other information (such as education or household size). Among the 2067 questionnaires, the majority came from South Africa (47.9%), while Italy, the United Kingdom, and Brazil accounted respectively for 20.3%, 17.8%, and 13.9% of the responses. Compared to the overall population, the sex ratio was unbalanced toward females: this is particularly evident for Brazil where male participants represented only 21.8% of the total. By contrast, the United Kingdom was the country with the most balanced sample in terms of sex with 53.0% of responses from female participants and 47.0% from male participants. The average participant age was 56.7 years (SD 15.6; maximum 93; median 61, IQR 47-68). This high value is attributed to the fact that our survey only targeted individuals over the age of 18 years and Facebook’s user base is diverse and comprises individuals of all ages, including those in the older demographic. Furthermore, older adults tend to participate more often in online surveys [35,36]. The average household size was 2.8 (SD 2.2, IQR 2-4). With regard to educational attainment, Italy and Brazil showed a majority of respondents with a secondary-level education (57.4% and 61.9%, respectively), while in South Africa and the United Kingdom, most of the respondents attained a university-level education (47.9% and 44.8%, respectively). For possible limitations of the sample composition, please refer to the Discussion.

**Table 2:** Breakdown of respondents by sex, age group, household size, and education for each surveyed country based on the unweighted sample (N=2067).

Variable			Italy (n=420), n (%)		South Africa (n=990), n (%)		United Kingdom (n=368), n (%)		Brazil (n=289), n (%)		Total (N=2067), n (%)
**Sex**											
	Female	261 (62.1)		670 (67.7)		195 (53.0)		226 (78.2)		1352 (65.4)	
	Male	159 (37.9)		320 (32.3)		173 (47.0)		63 (21.8)		715 (34.6)	
**Age (years)**											
	18-24	42 (10.0)		27 (2.7)		19 (5.2)		49 (17.0)		137 (6.6)	
	25-44	94 (22.4)		132 (13.3)		70 (19.0)		48 (16.6)		344 (16.6)	
	45-64	165 (39.3)		438 (44.2)		154 (41.8)		119 (41.2)		876 (42.4)	
	65+	119 (28.3)		393 (39.7)		125 (34.0)		73 (25.3)		710 (34.3)	
**Household size**											
	1	111 (26.4)		150 (15.2)		79 (21.5)		52 (18.0)		392 (19.0)	
	2	117 (27.9)		375 (37.9)		160 (43.5)		72 (24.9)		724 (35.0)	
	3-4	128 (30.5)		303 (30.6)		98 (26.6)		100 (34.6)		629 (30.4)	
	5+	28 (6.7)		135 (13.6)		26 (7.1)		47 (16.3)		236 (11.4)	
	No answer	36 (8.6)		27 (2.7)		5 (1.4)		18 (6.2)		86 (4.2)	
**Education**											
	Primary school	8 (1.9)		1 (0.1)		4 (1.1)		12 (4.2)		25 (1.2)	
	Secondary school	241 (57.4)		447 (45.2)		177 (48.1)		179 (61.9)		1044 (50.5)	
	University	159 (37.9)		474 (47.9)		165 (44.8)		79 (27.3)		877 (42.4)	
	Other	1 (0.2)		29 (2.9)		4 (1.1)		2 (0.7)		36 (1.7)	
	No answer	11 (2.6)		39 (3.9)		18 (4.9)		17 (5.9)		85 (4.1)	

### Behavioral Changes

**Figure 2:**
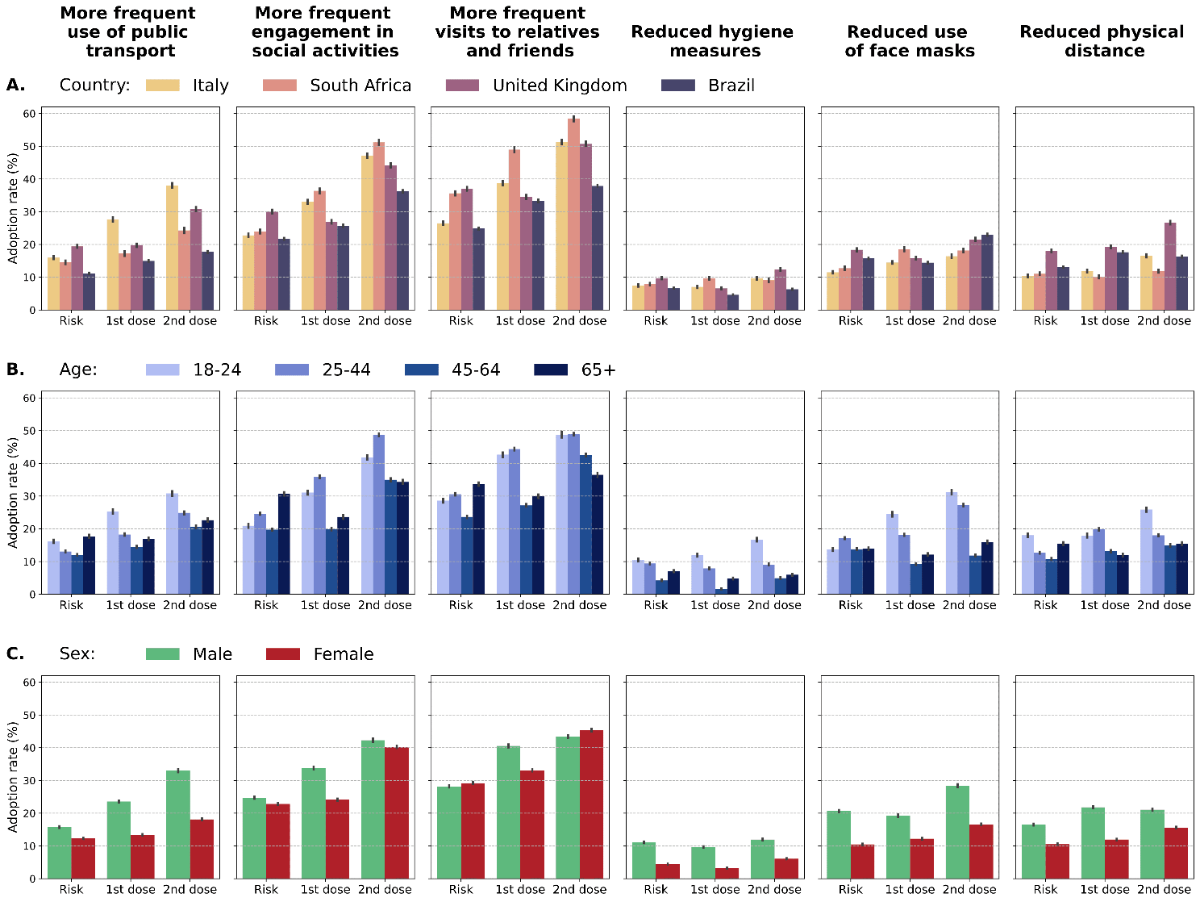
Adoption rate of behaviors after each stage of the vaccination campaign divided by country (A), age group (B), and sex (C). The three stages considered are: vaccination of people over 65 years or with comorbidities (Risk), after receiving the first vaccine dose (1st dose), and after receiving the second vaccine dose (2nd dose). Bar plots show mean values and 95% CIs as error bars.

Figure 2 shows the percentage of respondents by country (A), age (B), and sex (C) that reported a change in behaviors after the three key events of the vaccination campaign considered (vaccination of the population at risk and after the individual received the first and second doses). Across the board, the activity that was changed the most is “Visit relatives and friends more frequently”, followed closely by “More frequent engagement in social activities” such as going to restaurants and the cinema. By contrast, “Reduced hygiene measures” (eg, wash hands less often, use disinfectant gel less often) was the activity with the lowest percentage of change. Indeed, even after the second dose, the adoption rate exceeded 10% only in the United Kingdom. Similar findings can be observed for the two activities “Wear a face mask less often (where not mandatory)” and “Reduce the recommended physical distance (1 or 2 meters)“, for which the adoption rate was below 30% across all countries and events. For this reason, in the multivariate analysis, we focused our attention on social behaviors, defining behavioral change as the adoption of “Visit relatives and friends more frequently” or “Engage in social activities more frequently (eg, going to restaurants)”.

For all six activities considered, the adoption rate was higher after the second dose with respect to that after the first dose. Nonetheless, in Figure 2A, we can observe differences across countries. Indeed, Italy is the country where the population felt more comfortable to increase the use of public transport both after the first (28.2%) and the second (38.4%) dose, while in Brazil, the adoption rate was only 14.9% and 17.6%, respectively. Furthermore, participants from Brazil were less prone to change their social behaviors. For example, only 37.6% of respondents reported an increase in visits to relatives and friends after the second dose, while the equivalent figure for Italy, the United Kingdom, and South Africa was above 50%.

Figure 2B shows the adoption rate by age. Respondents under 25 years old were the most comfortable with reducing hygiene measures, the use of face masks, and physical distance and with using public transport more often, with a substantial increase in the adoption rate along with the progress of the vaccination campaign. By contrast, people over 45 years old were less prone to relax social behaviors, in particular after the first and second doses.

Finally, Figure 2C shows differences in adoption rates by sex. Male respondents were more comfortable to adopt almost every activity after all three stages of the vaccination campaign. The sole exception is represented by social behaviors for which, after the second dose, the adoption rate was quite similar for social activities in general (male: 42.0%; female: 40.3%), but was higher for female participants with respect to visits to relatives and friends (male: 43.1%; female: 45.3%).

**Figure 3:**
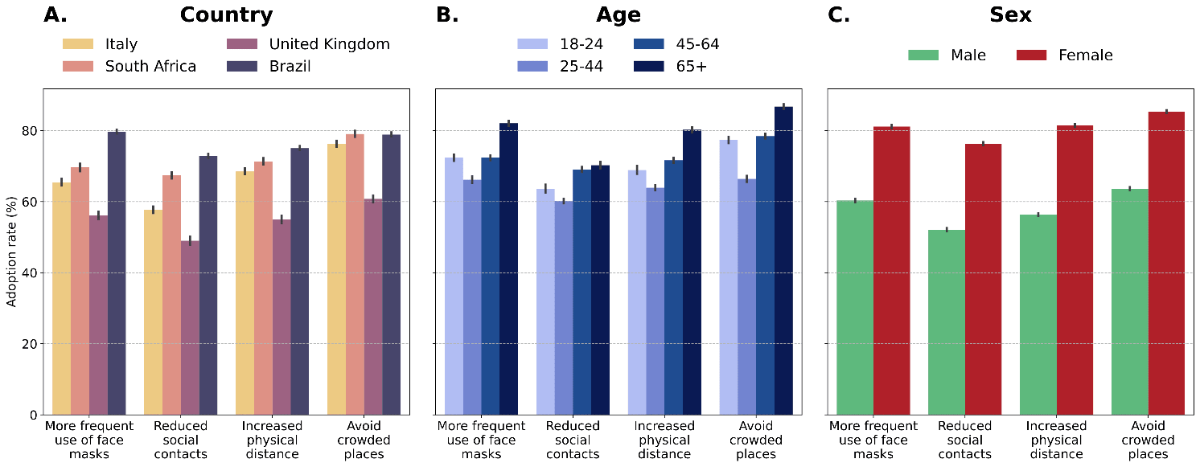
Adoption rate of preventive behaviors in case of a worsening of epidemiological conditions divided by country (A), age group (B), and sex (C). Bar charts show mean values as bars and bootstrapped 95% CIs as errors.

Figure 3 shows the adoption rate of preventive behaviors in the case of a potential worsening of epidemiological conditions by country (A), age group (B), and sex (C). Across the board, in the different cases considered, more than half of the participants reported that they would readopt COVID-safe behaviors in this hypothetical scenario. The United Kingdom showed the lowest adoption rate, exceeding 60% for avoiding crowded places only. When looking at the age breakdown in Figure 3A, we note that people over 65 years old would be more prone to readopt all the preventive behaviors. By contrast, the age groups with the lowest adoption rates were 25-44 and 18-24 years. We also noticed that reduction of social contacts was the preventive behavior with the lowest adoption rate, as it is likely perceived as more costly to adopt. Indeed, the adoption rate of this behavior was under 70% for all age groups. Finally, on average, female participants stated that they would adopt preventive behaviors more than male participants, with a difference of at least 20% in the adoption rate between sexes.

### Multivariate Analysis

**Figure 4:**
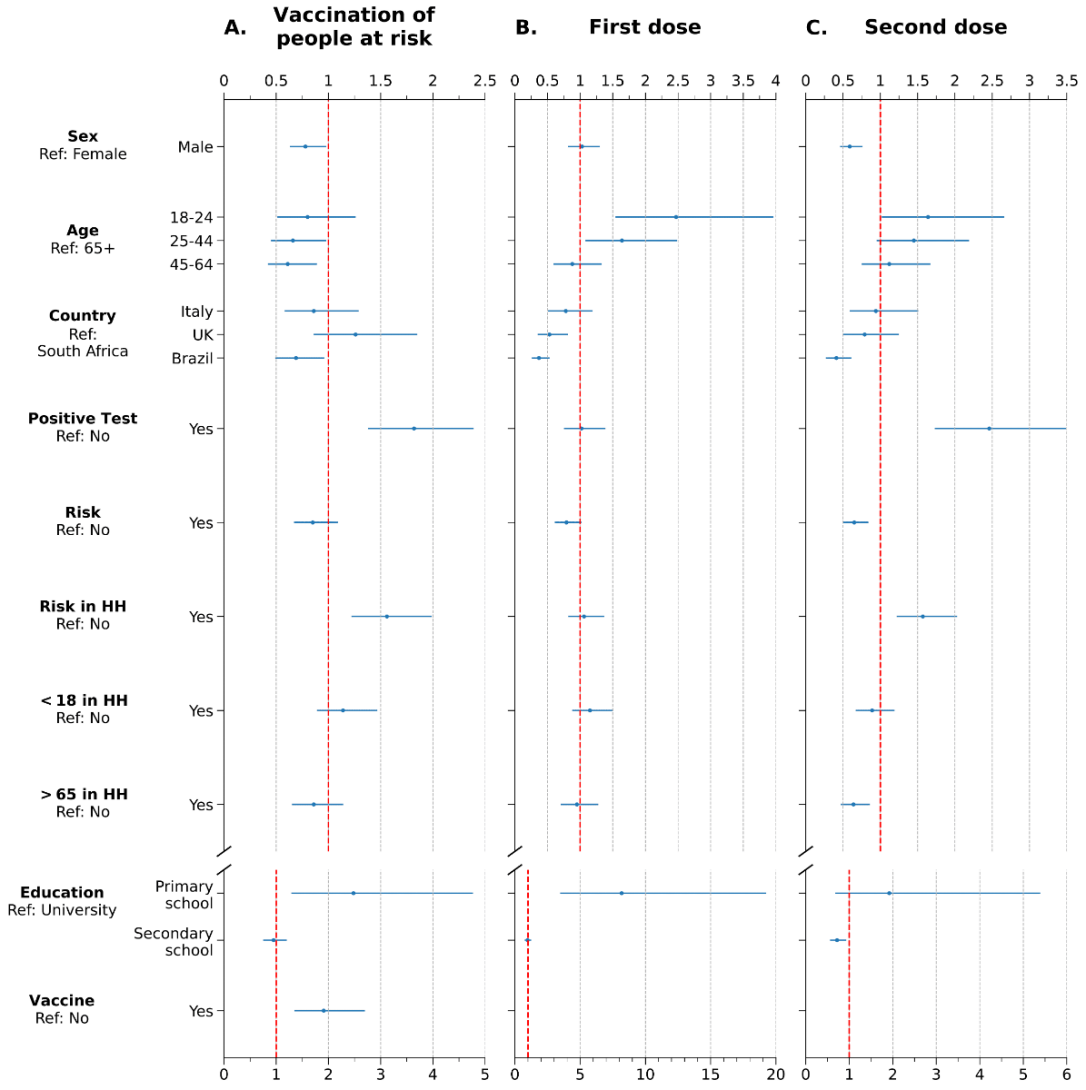
Odds ratios obtained from multivariate analysis of the survey responses related to behavioral changes after the vaccination of people over 65 years old or with comorbidities (A), after the first vaccine dose (B), and after the second vaccine dose (C). The binary outcome considered was 1 if there was a change in at least one of the two social behaviors and 0 otherwise. Details about the features are provided in Table 1. HH: household.

Figure 4 shows the ORs obtained from the multivariate analysis for changes in social behaviors after the three pivotal stages of the COVID-19 vaccination campaign. After the vaccination of older adults and people with comorbidities, having tested positive for COVID-19 was positively associated with a change in social behaviors (OR 1.82, 95% CI 1.38-2.39). This means that the odds of changing social behaviors for respondents that have been infected by COVID-19 are 1.82 times the odds for the rest of the population. A similar result was obtained for the feature risk in household, which was positively associated with a change in at least one of the two social behaviors (OR 1.56, 95% CI 1.22-1.99). Other features that were positively associated with the outcome were being vaccinated (OR 1.91, 95% CI 1.35-2.70) and a primary school–level education or lower (OR 2.48, 95% CI 1.29-4.77).

After the first dose, younger age groups engaged more frequently in social activities and visited relatives and friends more often. Indeed, the odds of a relaxation of social behaviors in the age group 18-24 was 2.47 times (95% CI 1.54-3.96) the odds of change in the age group 65+. The age group 25-44 and a primary school–level education or lower were also positively associated with an increase in social behaviors. Respondents from the United Kingdom and Brazil had lower odds to change social behaviors than respondents from South Africa.

After the second dose, having tested positive for COVID-19 (OR 2.46, 95% CI 1.73-3.49) and having people at risk within the household (OR 1.57, 95% CI 1.22-2.03) were positively associated with a change in social behavior. Conversely, the odds of these changes were lower for male respondents (OR 0.59, 95% CI 0.46-0.76), people at risk (OR 0.65, 95% CI 0.50-0.84), respondents from Brazil (OR 0.41, 95% CI 0.27-0.61), and those with a secondary school–level education (OR 0.72, 95% CI 0.56-0.93).

**Figure 5:**
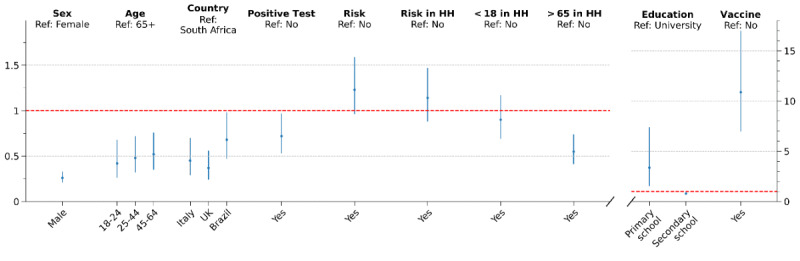
Odds ratios obtained from multivariate analysis of the answers related to behavioral changes in case of a worsening of epidemiological conditions. The binary outcome was 1, representing adoption of all four nonpharmaceutical interventions proposed, or 0 otherwise. Details about the features are provided in Table 1. HH: household.

Finally, in Figure 5, we report the ORs for the hypothetical scenario of a worsening of epidemiological conditions. In this case, the binary outcome was 1 if the participant would adopt all four NPIs proposed in the scenario and 0 otherwise. All age groups were less likely to adopt all the NPIs when compared to the 65+ years old group and the same tendency was found for male respondents with respect to female respondents. Other features showed a negative association, namely being a respondent from Italy, the United Kingdom, or Brazil; having tested positive for COVID-19; and having a household member aged over 65 years. In contrast, respondents with a primary school–level education or lower were more likely to adopt all four NPIs. However, the feature with the strongest positive association was the vaccination status. The odds of adopting all four NPIs in case of a worsening of epidemiological conditions for people who were vaccinated was 10.88 times higher (95% CI 6.96-17.01) than that for people who were not vaccinated.

## Discussion

### Principal Results

We found that a significant portion of participants relaxed NPIs during the vaccination campaign. In good accordance with the HBM, the two social behaviors were the activities that witnessed the greatest changes. Indeed, stronger relaxation of NPIs in social contexts can be explained considering their high perceived cost (ie, high perceived barriers in the HBM), which makes them difficult to be adopted for a long period. By contrast, the majority of individuals kept adopting personal activities such as hygiene measures and use of face masks throughout the duration of the vaccination campaign. These measures have indeed smaller costs associated and thus are easier to implement. This finding also shows how, after almost 2 years in a pandemic, face masks have become widely adopted and accepted, including in countries where they have rarely been used before, such as Italy.

We observed age- and sex-specific patterns. After the first and second vaccine doses, older respondents were far less inclined to relax their protective behaviors with respect to younger respondents. Older people also reported the highest adoption rate of NPIs in a hypothetical scenario of worsening of epidemiological conditions. This is consistent with the HBM, where perceived severity is one of the driving factors of the adoption of health behaviors. Indeed, older adults are at higher risk of severe symptoms from COVID-19 [37,38].

We found that female respondents reported smaller changes in NPIs with respect to male respondents. Consistently, in the multivariate analysis, we found that being female was positively associated with a change in social behaviors after the second vaccine dose. In the case of a worsening of epidemiological conditions, the difference was considerable: the adoption rate of NPIs was at least 15% higher among female than male respondents. These results are in line with previous research in the context of COVID-19 [29,39] or flu [40], where female participants were found to be more inclined to adopt preventive behaviors. However, it is important to note that while susceptibility to COVID-19 infection is similar for the two sexes, COVID-19 infection in male patients is associated with higher severity and fatality [41,42].

Two of the most important determinants for the drop of social NPIs were (1) having tested positive for SARS-CoV-2 and (2) having people at risk in the household. Indeed, both of these features were positively associated with a change in social behaviors after vaccination of the vulnerable population and after the second dose. This is probably due to the fact that after recovering from COVID-19, individuals are less worried about getting the virus again or spreading it and, as a consequence, they relax their social behaviors. Conversely, having people at risk in the household may impact the perceived threat of individuals and can lead them into adopting particularly careful behaviors in order to protect them. Therefore, after the vaccination of these vulnerable people or after their own vaccination, individuals may have felt safe (smaller perceived susceptibility and severity) to partially relax their protective behaviors. However, it is interesting that being at risk was negatively associated with a drop in social NPIs after the second dose. Therefore, while people around them felt safer to engage more frequently in social behaviors, individuals with a risk condition on average did not.

Finally, it is interesting to focus on the vaccination status. Obviously, this was not used as a feature to analyze change in behavior after the first and second doses since these questions were available only for vaccinated people. We found that being vaccinated was positively associated with a drop in social NPIs after the vaccination of those over 65 years and people with comorbidities. This can be explained by considering that this question was also available to all people who were vaccinated because of their risk condition (age or comorbidities). After being vaccinated, these people probably felt more protected and as a result relaxed their behaviors. By contrast, being vaccinated was positively associated with adoption of NPIs in case of a worsening of epidemiological conditions. This association was very strong: vaccinated people were 10.88 times more likely to adopt all the COVID-safe behaviors examined than nonvaccinated people. This is concerning because individuals that are not vaccinated, in addition to being less protected against COVID-19 from a pharmacological side, also lack behavioral protection as they would be less likely to adopt safer behaviors, exposing themselves to a higher risk of infection.

### Limitations

While the sensitivity analysis shown in Table S4 of Multimedia Appendix 2 indicated that our results are solid, there are limitations to our work. First, it is important to acknowledge that this study followed a cross-sectional design, which prevents us from drawing causal associations between exposures and outcomes. Furthermore, the cross-sectional nature of our survey poses limitations on directly comparing the adoption of behaviors before and after the vaccination campaign in our study. Second, responses obtained via online surveys administered on Facebook or other social media platforms are typically not representative of the general population [43-45]. To mitigate this issue, we carefully planned the data collection through Facebook advertisements targeted homogeneously across different demographic groups. The reliability of such targeting criteria for recruiting participants for survey research have been assessed in previous studies [46,47]. Moreover, we applied poststratification weights to correct for the remaining imbalances, at least in central observable characteristics such as age, sex, and region of residence. Confounders that were not included in the study may lead to residual confounding. Furthermore, we acknowledge possible self-selection bias of online survey respondents and underrepresentation of minorities. Another important element to consider is the language used for ads and surveys: English, Italian, and Portuguese. While these are the official languages of the four countries we focused on, this limited language availability may have caused underrepresentation of specific groups. This is especially true for South Africa, where English is the main language of only a fraction of the population despite being understood by more than half of the population and being the most common language in urban areas.

### Conclusions

NPIs have played a pivotal role in the first year of the COVID-19 pandemic, slowing the disease spread while vaccines were being developed and tested. Even after the start of vaccination campaigns, NPIs remained essential [14]. Indeed, due to limited supplies (especially in low- and middle-income countries [35,48-52]) and unprecedented logistic challenges, NPIs were key to mitigating the disease burden as vaccinations progressed [14-18]. Nevertheless, the milestone marked by the arrival of effective vaccines, in a background of pandemic fatigue, might have affected risk perception of segments of the population, inducing a reduction in NPIs compliance [19]. Several modeling efforts highlighted the potential negative effects of such a phenomenon, whereas empirical supporting evidence remains limited. Here, we tackled this limitation by investigating whether individuals relaxed behaviors during the vaccination campaign using an online survey administered via Facebook, collecting more than 2000 responses across four countries. Moreover, to understand the role played in the relaxation of NPIs by different social and demographic characteristics, we performed a multivariate analysis focusing on the drop of NPIs in social contexts. We showed a significant relaxation of COVID-19 safe behaviors, in particular social activities, and we found that the main determinants of these changes are generally connected to shifts in perceived risk. Therefore, great caution should be taken during a mass vaccination campaign such as that experienced in the last few years during the COVID-19 pandemic. Indeed, spontaneous relaxation of NPIs by the population can jeopardize the incredible benefits of the immunization campaign; although not immediately evident, these effects are visible over the medium term, especially in challenging and emergency contexts. For this reason, it is extremely important for policy makers to maintain high compliance with NPIs in the first phase of a vaccination campaign through targeted actions and efficient communication.

Ultimately, our results can also be used to inform and design more advanced, data-driven epidemic-behavioral mathematical models that are capable of more accurately capturing the spread of the virus, the behavioral reaction of individuals, and the progress of the vaccination campaign.

## Appendix

Multimedia Appendix 1 English version of the questionnaire.

Multimedia Appendix 2 Scheme of the Facebook advertising campaign (Figure S1), macro/micro regions of each country (Table S1), detailed respondent selection criteria, distribution of "no answer" or "not applicable" responses (Table S2), variance inflation factors for analysis features (Table S3), robustness analysis (Table S4).

## Abbreviations

GLM: generalized linear model
HBM: Health Belief Model
NPI: nonpharmaceutical intervention
OR: odds ratio
VOC: variant of concern
