# Supplementary material for: Behavioral Changes Associated With COVID-19 Vaccination: Cross-National Online Survey
Source: J Med Internet Res. 2023 Oct 31;25:e47563. doi: 10.2196/47563 (PMC10646669; doi:10.2196/47563)
Supplement: Multimedia Appendix 1 [file jmir_v25i1e47563_app1.pdf]

Multimedia Appendix 1
English version of the questionnaire

Introduction

Vaccination and behavior
Welcome to our Covid-19 vaccines and behaviors Survey.
This survey is part of a research project promoted by ISI Foundation (www.isi.it), a private research institution in Turin, Italy, in collaboration with Greenwich University (London, UK) and the Max Plank Institute for Demographic Research (Rostock, Germany).
The goal of the research is to study the link between individual preventive measures and vaccination against COVID-19. Your participation is crucial for our study.
The survey is directed at all people who are 18 years old or older, and it will take few minutes to complete. Your participation is completely voluntary, you can stop participating at any time. In case you are not comfortable answering a particular question, you have the option to select "No answer". Participants' data will be treated anonymously, and we will not ask for identifying information.
If you have any questions about this research study, please write to behaviorsurvey@isi.it
To take vision of our data protection policy you can press the 'Show Policy' button in the box below.
Tick the box below and press 'Next' to continue.
I am willing to participate in this survey, I am at least 18 years old, and I have read the data protection policy.

Socio-demographic

- Q0 - What is your gender?
  - Q0.1 Male
  - Q0.2 Female
  - Q0.3 Other
- Q1 - What is your birth year? DATE/TIME BOX (yyyy)
- Q2 - In which country do you currently live? DROPDOWN MENU
- Q3 - In which city do you live? TEXT BOX
- Q4 - What is your highest level of education?
  - Q4.1 No formal education
  - Q4.2 Primary school

- Q4.3 Secondary school
- Q4.4 University-level education (e.g. bachelor's degree, master's degree)
- Q4.5 Postgraduate degree (e.g. Ph.D., Medical Doctorate)
- Q4.6 Other

- Q5 - Have you ever tested positive for COVID-19?
  - Q5.1 - Yes
  - Q5.2 - No
  - Q5.3 - I don't know

Vaccine-Behaviour

- Q6 - Do you (or someone in your household) have a medical condition that is a risk factor for COVID-19 (examples are cancer, heart conditions, respiratory chronic diseases, immunocompromised state, obesity)?
  - Q6.1 Yes, me
  - Q6.2 Yes, someone in my household
  - Q6.3 Yes, both
  - Q6.4 No
  - Q6.5 I don't know

If Q6.2 is True or Q6.3 is True

- Q6b - After the vaccination of people in your household who have a risk factor for COVID-19, did you (or would you) feel more comfortable doing the following activities? (answers: "1 - Definitely not", "2 - No", "3 - Neither yes nor no", "4 - Yes", "5 - Definitely yes", or "Not applicable"):
  - Q6b.1 Use public transport more frequently
  - Q6b.2 Engage in social activities more frequently (e.g. going to restaurants)
  - Q6b.3 Visit relatives and friends more frequently
  - Q6b.4 Reduce hygiene measures (e.g., wash your hands less often, use disinfectant gel less often)
  - Q6b.5 Wear a face mask less often (where not mandatory)
  - Q6b.6 Reduce the recommended physical distance (1 or 2 meters) from other people

- Q7 - How many people, INCLUDING YOU, are currently living in your household?
  - Q7.1 Children under 18 years old DROPDOWN MENU (0-20)
  - Q7.2 Adults from 18 to 64 years old DROPDOWN MENU (0-20)
  - Q7.3 Adults 65 years old or older DROPDOWN MENU (0-20)

If Q7.3 $>$ 0 :

- Q7b - After the vaccination of people over 65 in your household, did you (or would you) fell more comfortable doing the following activities? (answers: ”1 - Definitely not”, ”2 - No”, ”3 - Neither yes nor no”, ”4 - Yes”, ”5 - Definitely yes”, or ”Not applicable”):
  - Q7b.1 Use public transport more frequently
  - Q7b.2 Engage in social activities more frequently (e.g. going to restaurants)
  - Q7b.3 Visit relatives and friends more frequently
  - Q7b.4 Reduce hygiene measures (e.g., wash your hands less often, use disinfectant gel less often)
  - Q7b.5 Wear a face mask less often (where not mandatory)
  - Q7b.6 Reduce the recommended physical distance (1 or 2 meters) from other people
- Q8 - Have you had a COVID-19 vaccination?
  - Q8.1 Yes
  - Q8.2 No, not yet but I want to do it
  - Q8.3 No, I can’t do it (because of allergies, medical conditions...)
  - Q8.4 No, I don’t want/need the COVID-19 vaccination
  - Q8.5 I don’t know

  If Q8.1 is True:
- Q8b - How many COVID-19 vaccinations have you received?
  - Q8b.1 One vaccination or dose
  - Q8b.2 Two vaccinations or doses
  - Q8b.3 Three vaccinations or doses
  - Q8b.4 I don’t know

  If Q8.1 is True:
- Q8c - Which vaccine did you receive?
  - Q8c.1 Pfizer–BioNTech
  - Q8c.2 Moderna
  - Q8c.3 Vaxzevria (AstraZeneca)
  - Q8c.4 Janssen (Johnson & Johnson)
  - Q8c.5 CoronaVac (Sinovac)
  - Q8c.6 Other

  If Q8.1 is True:
- Q8d – In which month did you receive the FIRST dose of the vaccine? DATE/TIME BOX (mm/yyyy)

  If Q8.2 is True or Q8.5 is True:

- Q9a - If you were already vaccinated against COVID-19, would you feel more comfortable doing the following activities? (answers: "1 - Definitely not", "2 - No", "3 - Neither yes nor no", "4 - Yes", "5 - Definitely yes", or "Not applicable"):
  - Q9a.1 Use public transport more frequently
  - Q9a.2 Engage in social activities more frequently (e.g. going to restaurants)
  - Q9a.3 Visit relatives and friends more frequently
  - Q9a.4 Reduce hygiene measures (e.g., wash your hands less often, use disinfectant gel less often)
  - Q9a.5 Wear a face mask less often (where not mandatory)
  - Q9a.6 Reduce the recommended physical distance (1 or 2 meters) from other people

  If Q8.1 is True and Q8b.1 or Q8b.2 or Q8b.3 is True:

- Q9b - After receiving the FIRST dose of vaccine, did you feel more comfortable doing the following activities? (answers: "1 - Definitely not", "2 - No", "3 - Neither yes nor no", "4 - Yes", "5 - Definitely yes", or "Not applicable"):
  - Q9b.1 Use public transport more frequently
  - Q9b.2 Engage in social activities more frequently (e.g. going to restaurants)
  - Q9b.3 Visit relatives and friends more frequently
  - Q9b.4 Reduce hygiene measures (e.g., wash your hands less often, use disinfectant gel less often)
  - Q9b.5 Wear a face mask less often (where not mandatory)
  - Q9b.6 Reduce the recommended physical distance (1 or 2 meters) from other people

  If Q8.1 is True and Q8b.2 or Q8b.3 is True:

- Q9c - After receiving the SECOND dose of vaccine against COVID-19, did you fell more comfortable doing the following activities? (answers: "1 - Definitely not", "2 - No", "3 - Neither yes nor no", "4 - Yes", "5 - Definitely yes", or "Not applicable"):
  - Q9c.1 Use public transport more frequently
  - Q9c.2 Engage in social activities more frequently (e.g. going to restaurants)
  - Q9c.3 Visit relatives and friends more frequently
  - Q9c.4 Reduce hygiene measures (e.g., wash your hands less often, use disinfectant gel less often)
  - Q9c.5 Wear a face mask less often (where not mandatory)
  - Q9c.6 Reduce the recommended physical distance (1 or 2 meters) from other people

  If Q8.2 is True or Q8.3 is True or Q8.4 is True:

- Q9d - After the beginning of the vaccination campaign in your country among the general population, did you fell more comfortable doing the following activities? (answers: "1 - Definitely not", "2 - No", "3 - Neither yes nor no", "4 - Yes", "5 - Definitely yes", or "Not applicable"):
  - Q9d.1 Use public transport more frequently

  - Q9d.2 Engage in social activities more frequently (e.g. going to restaurants)
  - Q9d.3 Visit relatives and friends more frequently
  - Q9d.4 Reduce hygiene measures (e.g., wash your hands less often, use disinfectant gel less often)
  - Q9d.5 Wear a face mask less often (where not mandatory)
  - Q9d.6 Reduce the recommended physical distance (1 or 2 meters) from other people

- Q10 - Which fraction of the population should be vaccinated before starting to relax behaviours (examples are wearing masks less often, engaging more frequently in social activities, taking public transports more often)?
  - Q10.1 Less than 25% targeted to the vulnerable (elderly and people with comorbidities)
  - Q10.2 Less than 25%
  - Q10.3 Between 25% and 50%
  - Q10.4 Between 50% and 75%
  - Q10.5 Above 75%

- Q11 - After the vaccination of elderly (people over 65) and people with comorbidities, did you feel more comfortable doing the following activities? (answers: ”1 - Definitely not”, ”2 - No”, ”3 - Neither yes nor no”, ”4 - Yes”, ”5 - Definitely yes”, or ”Not applicable”):
  - Q11.1 Use public transport more frequently
  - Q11.2 Engage in social activities more frequently (e.g. going to restaurants)
  - Q11.3 Visit relatives and friends more frequently
  - Q11.4 Reduce hygiene measures (e.g., wash your hands less often, use disinfectant gel less often)
  - Q11.5 Wear a face mask less often (where not mandatory)
  - Q11.6 Reduce the recommended physical distance (1 or 2 meters) from other people

- Q12 - In case of a worsening of the epidemiological conditions (e.g. increase in deaths) in the future, would you (answers: ”1 - Very unlikely”, ”2 - Unlikely”, ”3 - Neutral”, ”4 - Likely”, “5 - Very likely” or “not applicable”):
  - Q12.a Wear a face mask more frequently (where not mandatory)
  - Q12.b Reduce social contacts
  - Q12.c Keep a higher physical distance from other people
  - Q12.d Avoid crowded places

  If Q8.1 or Q8.2 is true and Q8b.3 is False

- Q13 - Would you be willing to receive a THIRD dose of the vaccine, in case it becomes available in the near future?
  - Q13.1 Yes
  - Q13.2 No
  - Q13.3 I don’t know

Conclusion

Thank you for participating!
For any additional information please write to behaviorsurvey@isi.it
