# Supplementary material for: Behavioral Changes Associated With COVID-19 Vaccination: Cross-National Online Survey
Source: J Med Internet Res. 2023 Oct 31;25:e47563. doi: 10.2196/47563 (PMC10646669; doi:10.2196/47563)
Supplement: Multimedia Appendix 2 [file jmir_v25i1e47563_app2.pdf]

Multimedia Appendix 2

S1 Facebook campaigns

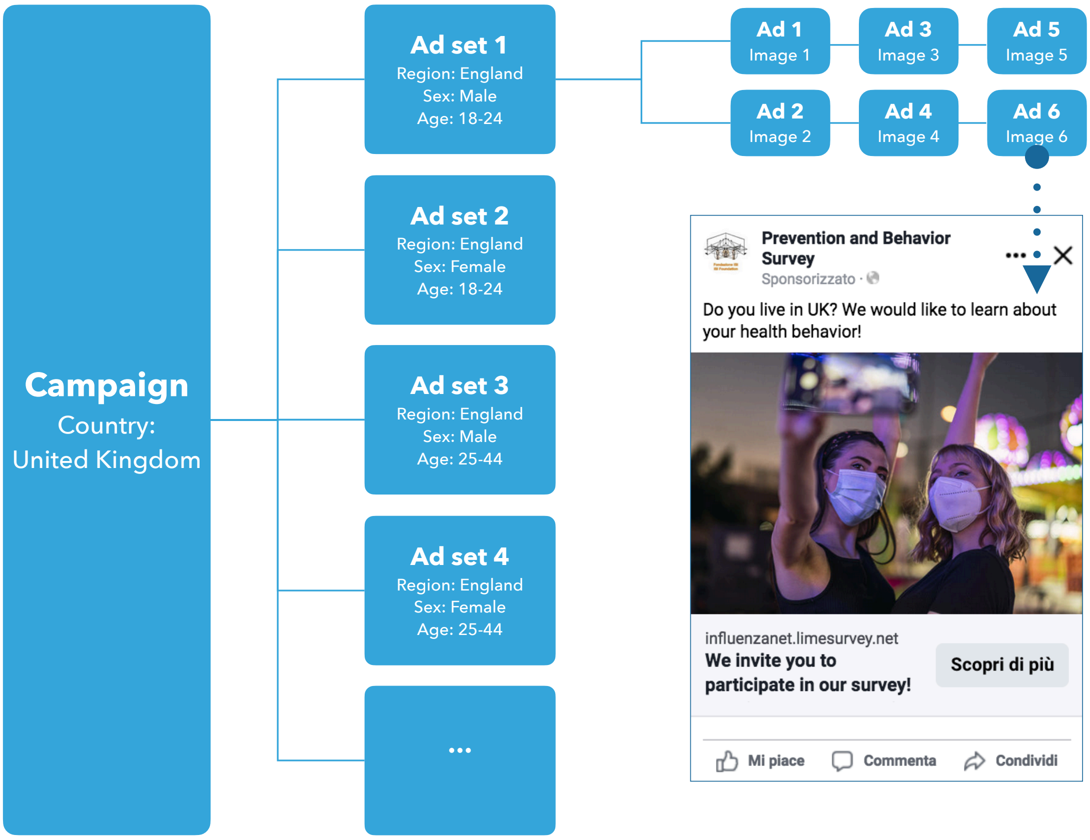

Figure S1: Scheme of the structure of the Facebook advertising campaign used in United Kingdom with an example of ad. Adapted from [28] and [29]

Facebook allows to target a wide public selecting the desired characteristics (age, gender, region, interests...). Each campaign is structured in 3 levels: a) the campaign level, at which it is possible to set the goals of the campaign (such as generating clicks, generating contacts, generating sells, etc.), b) the ad set level, at which it is possible to specify the targets (each ad set has its own targets),

c) the ad level, at which it is possible to specify the text and the images to use for each ad. As mentioned in the main text, we created a campaign for each country and each campaign contained several ad sets, each targeting a specific group of the population according to sex (Male and Female), age (18-24, 25-44, 45-64, 65+) and macro-region (details in Table S1). The number of ad sets varied from 32 to 40. Finally, each ad sets contained 6 ads, which differed from each other only by the image used. In Figure S1 it is possible to see a scheme of the structure of the Facebook campaign we used for United Kingdom.

<table>
<tr><th>Country</th><th>Macro-region</th><th>Micro-region</th></tr>
<tr><td rowspan="5">Italy</td><td>Central</td><td>Lazio, Marche, Toscana, Umbria</td></tr>
<tr><td>Insular</td><td>Sardegna, Sicilia</td></tr>
<tr><td>Northeast</td><td>Emilia-Romagna, Friuli-Venezia Giulia, Trentino-Alto Adige, Veneto</td></tr>
<tr><td>Northwest</td><td>Liguria, Lombardia, Piemonte, Valle d'Aosta</td></tr>
<tr><td>South</td><td>Abruzzo, Basilicata, Calabria, Campania, Molise, Apulia</td></tr>
<tr><td rowspan="5">United Kingdom</td><td>England</td><td>East Midlands, East of England, North East, North West, South East, South West, West Midlands, Yorkshire and The Humber</td></tr>
<tr><td>London</td><td>London</td></tr>
<tr><td>Northern Ireland</td><td>Northern Ireland</td></tr>
<tr><td>Scotland</td><td>Scotland</td></tr>
<tr><td>Wales</td><td>Wales</td></tr>
<tr><td rowspan="5">Brazil</td><td>Central-West</td><td>Goiás, Mato Grosso, Mato Grosso do Sul, Distrito Federal</td></tr>
<tr><td>North</td><td>Acre, Amapá, Amazonas, Pará, Rondônia, Roraima, Tocantins</td></tr>
<tr><td>Northeast</td><td>Alagoas, Bahia, Ceará, Maranhão, Paraíba, Pernambuco, Piauí, Rio Grande do Norte, Sergipe</td></tr>
<tr><td>South</td><td>Paraná, Rio Grande do Sul, Santa Catarina</td></tr>
<tr><td>Southeast</td><td>Espírito Santo, Minas Gerais, Rio de Janeiro, São Paulo</td></tr>
<tr><td rowspan="4">South Africa</td><td>Central-East</td><td>KwaZulu-Natal, Free State</td></tr>
<tr><td>Gauteng</td><td>Gauteng</td></tr>
<tr><td>North</td><td>Mpumalanga, Limpopo, North West</td></tr>
<tr><td>Southwest</td><td>Eastern Cape, Northern Cape, Western Cape</td></tr>
</table>

Table S1: Subdivision in macro and micro regions of each country included in the analysis.

Due to the limited number of active ads that are available at the same time for each account, we could promote our survey only in one country at a time. We started with Italy from November 26, 2021 to December 4, 2021, then South Africa from December 4, 2021 to December 10, 2021, next United Kingdom from December 10, 2021 to December 16, 2021 and finally Brazil from December 16, 2021 to December 21, 2021. Previous studies showed that the day of the week doesn't affect the answers to the survey and, thus, time duration of campaigns varies from 5 days (Brazil) to 8 days (Italy). The goal selected for our ads was "Traffic" because we wanted Facebook users to click on the link to our survey, visible in each ad. However, Facebook algorithms tend to show ads to people who are most likely to click on the link. In this way the representativeness is sacrificed in favour of higher numbers of clicks. While one of our goal was to get as many surveys filled as possible, we wanted at the same time to obtain answers from the widest public possible. This is the reason why, we created between 32 and 40 different ad groups for each campaign, stratifying for sex, age group

and macro-regions within countries.

S2 Respondent selection

As mentioned in the main text, for the analysis on behavioral changes we have included only participants who reported their country, age and sex. However, certain questions were not accessible to all the participants or participants may not have answered to some of them. The question related to behaviors after the vaccination of over 65 years old and people with comorbidities and the question on the hypothetical scenario of a worsening of epidemiological conditions were both accessible to everyone and, respectively, 1915 and 2008 respondents replied to them. On the other hand, questions on first and second dose were accessible only to those who reported to have received at least one or two doses of the vaccine for SARS-CoV-2 and we were received answers from, respectively, 1601 and 1480 respondents. Finally, a participant may have reported how comfortable they felt to relax their hygiene measure but may not have answered about visits to parents and friends. In Table S2 we reported the numbers of respondents who did not answer about each activity for each event of the vaccination campaign. It can be seen, that the number are extremely small, and they never account for more than 3.5 % of the total answers and, for this reason, we decided not to exclude them from the analysis. The only exception is represented by public transportation. Indeed, several respondents weren't using them neither before nor after the events examined and for this reason they chose the option "Not applicable". Despite they may account for an higher percentage of the total, we included them in our analysis as we did for the other activities.

	Vaccination of people at risk	First dose	Second dose
More frequent use of public transport	281 (14.7%)	257 (16.1%)	228 (15.4%)
More frequent engagement in social activities	63 (3.3%)	37 (2.3%)	30 (2.0%)
More frequent visits to relatives and friends	42 (2.2%)	32 (2.0%)	27 (1.8%)
Reduced hygiene measures	34 (1.8%)	21 (1.3%)	21 (1.4%)
Reduced use of face masks	51 (2.7%)	25 (1.6%)	22 (1.5%)
Reduced physical distance	42 (2.2%)	22 (1.4%)	15 (1.0%)

	Worsening of epidemiological conditions
More frequent use of face masks	57 (2.8%)
Reduced social contacts	30 (1.5%)
Increased physical distance	32 (1.6%)
Avoid crowded places	19 (0.9%)

Table S2: Number (and percentage) of "No answer" or "Not applicable" for each activity and each question.

S3 Variance Inflation Factor

In order to check how independent are the features that we selected for the multivariate analysis, we computed the Variance Inflation Factor (VIF). The VIF quantifies the severity of multicollinearity between features and it ranges from 1 upwards. A VIF equal to 1 indicates the absence of correlation, and the higher it gets and the more severe is the multicollinearity. In general, it is often used a cutoff of 5. In Table S3, it is possible to see that the multicollinearity is extremely low (close to 1) for every feature with the sole exception of age group 25-44, age group 45-64 and >65 in HH for which we have slightly higher values (around 2).

Features		VIF
Sex (Ref: Female)	Male	1.07
Age (Ref: 65+)	18-24	1.40
	25-44	1.99
	45-64	2.27
Country (Ref: South Africa)	Italy	1.22
	United Kingdom	1.17
	Brazil	1.20
Positive Test (Ref: No)	Yes	1.02
Risk (Ref: No)	Yes	1.04
Risk in HH (Ref: No)	Yes	1.05
<18 in HH (Ref: No)	Yes	1.15
>65 in HH (Ref: No)	Yes	1.88
Education (Ref: University)	Primary school	1.05
	Secondary school	1.05
Vaccine (Ref: No)	Yes	1.08

Table S3: Variance Inflation Factor for each feature included in the analysis.

S4 Model and odds ratio robustness

In this section we tested the robustness of our results. First of all, we checked the performance of several models. In addition to the logistic regression with weights, we also tried with Support Vector Machine, Random Forest and XGBoost. The latter models, are based on more complex algorithms which may offer higher performance but at the expense of a smaller interpretability. The performance of the four models gave similar results in terms of prediction and, for this reason, we chose to use the logistic regression with weights, which was the one that allowed the highest explainability of the features.

Furthermore, we verified that the odds ratios obtained were robust. In order to do so, we computed the multivariate analysis again, removing one or more of the previously included features to see if the odds ratios of the other features were affected. In Table S4 we reported the results achieved for the second dose but similar results are obtained for the other events of the vaccination campaign. The first two columns are the odds ratios obtained including all the features and the corresponding p-values. *Mean 1* and *Std 1* are, respectively, mean and standard deviation of the

odds ratio for the corresponding feature obtained by removing each one of the other features, one at a time, and performing the analysis. For instance, the value of 0.59 reported in *Mean 1* for Sex_Male is the mean of the odds ratio obtained from multivariate analysis without the Age feature, the one obtained without the Country feature and so on. In column *% 1* we also reported how many of these odds ratios were significant (p-value smaller than 0.05). It can be seen that all the odds ratios that were significant when all the features were present, are barely affected by the absence of one feature: the mean value is very close to the original OR and the standard deviation is small. Indeed, values of *% 1* equal to 100% tell us that every odds ratio obtained removing one of the other feature is significant. A similar process has been applied by removing two features at a time (with results reported in columns *Mean 2*, *Std 2* and *% 2*) and three features at a time (columns *Mean 3*, *Std 3* and *% 3*. Similar as before, the absence of two or three features in the multivariate analysis doesn't impact the odds ratio of significant features. Indeed, for these latter, mean values remain close to ORs with all the features, standard deviations are small and a 100% of the odds ratio computed are significant.

	OR	P-value	Mean 1	Std 1	% 1	Mean 2	Std 2	% 2	Mean 3	Std 3	% 3
Sex_Male	0.59	<0.001	0.59	0.010	100.0	0.60	0.014	100.0	0.60	0.017	100.0
Age_18-24	1.64	0.042	1.67	0.199	61.5	1.68	0.263	69.2	1.66	0.304	64.7
Age_25-44	1.45	0.082	1.48	0.199	30.8	1.50	0.254	48.7	1.50	0.285	56.6
Age_45-64	1.12	0.573	1.13	0.152	7.7	1.12	0.195	12.8	1.11	0.221	15.7
Country_Italy	0.94	0.808	1.02	0.289	7.7	1.09	0.363	15.4	1.15	0.405	23.1
Country_UK	0.79	0.316	0.85	0.235	7.7	0.91	0.299	15.4	0.96	0.338	22.7
Country_Brazil	0.41	<0.001	0.42	0.026	100.0	0.42	0.032	100.0	0.43	0.038	100.0
Positive Test	2.46	<0.001	2.46	0.058	100.0	2.46	0.075	100.0	2.46	0.086	100.0
Risk	0.65	<0.001	0.65	0.010	100.0	0.65	0.014	100.0	0.65	0.016	100.0
Risk in HH	1.57	<0.001	1.57	0.051	100.0	1.56	0.064	100.0	1.55	0.074	100.0
<18 in HH	0.89	0.434	0.90	0.031	0.0	0.91	0.048	0.0	0.92	0.065	0.0
>65 in HH	0.64	0.003	0.63	0.037	100.0	0.62	0.046	100.0	0.62	0.050	99.7
Edu_Primary school	1.92	0.218	1.92	0.203	0.0	1.92	0.269	0.0	1.92	0.316	1.0
Edu_Secondary school	0.72	0.011	0.71	0.027	100.0	0.70	0.034	100.0	0.69	0.040	100.0

Table S4: Odds ratio without features.
